# Supplementary material for: Metastatic colorectal adenocarcinoma tumor purity assessment from whole exome sequencing data
Source: PLoS One. 2023 Apr 6;18(4):e0271354. doi: 10.1371/journal.pone.0271354 (PMC10079084; doi:10.1371/journal.pone.0271354)
Supplement: Attachment — Submitted filename: Response to Reviewers.docx [file pone.0271354.s001.docx]

**Re manuscript resubmission: Metastatic colorectal adenocarcinoma purity assessment from whole exome sequencing**

Case 07521338

**Response to reviewer comments**

**Reviewer #1:**

1. The platforms of tumor purity estimation in this study are relatively old. Please explain why they applied these three methods among many others.

Doi: 10.1093/bioinformatics/btz406. [1]

DOI: 10.1200/PO.20.00016 JCO [2] (2020)

Response: Thank you for this comment. We believe the three tools we selected represent the available in silico tools that utilize WES as the input data very well. The two evaluation criteria we used when selecting candidate in silico tools was

1) compatibility with our dataset because different in silico tools use different types of input data, and
2) comprehensive documentation because developers often fail to provide sufficient supporting documentation for new bioinformatics analysis packages.

Among the many tumor purity tools available for WES data, our chosen three tools were the most compatible with our data. We did use ABSOLUTE (2012), which is one of the tools also used in the systematic study (source #2 from the reviewer, reference #7 in our manuscript). Although ABSOLUTE tool is relatively old, it has been used in previous assessments of tumor purity tools including [2] and (https://www.ncbi.nlm.nih.gov/pmc/articles/PMC4794615/ - **6 in our citations). We used this tool as a means to compare our assessment to that of previous reports of tumor purity estimation tools. All other tools mentioned in [2] were not used due to data compatibility issues such as incompatible system requirements and file formatting conflicts from upstream analysis. Further rationale for tool selection is as follows:

Sequenza (2015) was chosen for its compatibility with our data. In addition, it is a well cited and well documented tool that produces a comprehensive set of visualizations for the user to understand the resulting purity estimate. Sequenza provided matched normal tumor purity support that served as a third avenue of comparison to get a comprehensive estimate of our tumor purities.

PureCN (2016) was chosen because it was the only tool compatible with our WES data from the patient tumor but not from matching normal DNA (= ‘tumor-only’ samples). It also has a variety of visualizations produced from its analysis pipeline. Additionally, the tool is well cited and well documented. We used this tool to estimate purities for all our tumor only samples. This was the only tool we could find that had tumor-only support compatible with our data. Although we could not compare its performance against other in silico tumor purity methods, using this tool gave us a fair estimate of tumor purity where otherwise we would have no estimate at all.

2. Organoids were used as a control of mostly pure cancer cells in this study. Since it is difficult to understand the context, it should be briefly noted in abstract why the organoids were used.

Response: Thank you for this useful remark, we have modified the abstract accordingly, line 34. As stated in the comment above, organoids were used as a control to help assess the *in silico* tools performance as they represent high tumor purity samples.

3. Method for organoid preparation and culture should be described more in detail, especially the timing of the sampling after preparation or culture. Longer culture period might select the cancer cells adapted to the culture conditions.

Response: We have expanded the organoid culture section under Materials and methods, line 103 and following paragraph.

Cancer cell selection is an excellent point and one that we discussed as well. Initially we intended only to use organoids generated directly from patient tumors (PDOs), meaning without any xenograft step, as we reasoned that passaging in animals may also select for cancer cell subclones. Unfortunately, not all patient tumors yielded organoids with this method. Instead, some of the tumor specimens failed to produce PDOs but did take as a PDX, and vice versa. All organoids were low passage, both PDOs and PDXOs, when DNA was extracted (p < 5). One notable exception was CR1107 PDOs, with a passage number of 16.

We agree that selection is likely going to happen in long term cancer cell culture, regardless of the method. We did find high concordance (>90%) between the parent tumors and the matching organoids when comparing all SNPs (both non-pathogenic and pathogenic). But more importantly, pathogenic mutations found in the variant analysis were identical between the parent tumors and organoids (apart from CR1106, more on this below).

4. The timings of the operations after chemotherapy should be shown.

Response: Unfortunately, we do not have access to information about the timing of the last chemotherapy dose each patient received before their hepatectomies. We do mention the need for more in depth analysis in the discussion, line 351.

However, since we are comparing several different computational methods using the same WES data, our findings of varying VAF calls and tumor purity percentages *per patient sample* should not be influenced by prior treatment.

5. Histopathological analysis should be described more in detail, since it will make a big difference when the estimation is based on the tumor/stromal ‘area’ or ‘cells’. It would be better to show representative HE images along with the instruction of the way of the histopathological analysis; each area, each cell, and so on. It would be better to perform the histopathological analysis at multiple sites to show the heterogenous cancer cell purity in one tumor.

Response: Unfortunately, we don’t have access to samples from multiple sites of the tumors. However, we now included an HE image (Fig3) to better illustrate how the pathologist assessment was performed. Additionally, as suggested, the pathologist now specifically estimated the proportion of tumor cells within the tumor area (Fig3, table 2).

6. P3L42 Since the molecular/computational and histopathological estimation are not consistent in this study, the conclusion of the abstract, just saying combination of the analyses is important is not appropriate.

Response: We respectfully disagree. As stated in the comments from reviewer#2, our cohort size is rather small. Thus, we are hesitant to universally recommend one purity estimation method over another. Rather, we believe a combination of methods is likely to produce a better purity estimate over relying on any individual approach. Due to other features that pathologists evaluate (necrosis, etc), we find that there is still a large need for this assessment, and have altered the abstract to reflect the multifactorial decision making that should go into tumor purity estimates.

7. P6L91-93 Please describe the relationship between snap frozen samples and fresh samples. Were they from the same samples or from different sites?

Response: Thank you for this useful comment. We have modified this description in the manuscript, line 92. One tumor sample was divided into three parts where one piece was snap frozen in liquid nitrogen for DNA extractions, a second (fresh) piece was used for generating a xenograft and a third (fresh) piece was used for generating patient-derived organoids (PDOs).

8. P10L180 ‘tumor’ is better to be ‘cancer cells’

Response: Thank you, pathologist has now analyzed the tumor purity as percentage of tumor cells within tumor area, line 187.

9. P11-P12 The purity estimation results are described as ‘low’ and ‘high’. Please make the criteria of low and high clearer.

Response: Based on the TCGA guidelines, we consider purity <60% low and >60% high. We have included this in the manuscript, line 206-207.

10. Table 2 Since this table is not in comparison with the other types of estimation, it is difficult to interpret the data for a reader. It would be better to show the tumor purity at least also in figure 1. It seems like that the histopathological estimation is performed by areas but not cells. If so, it is not surprising that the estimation by ‘area’ are not consistent with the estimation by ‘cells’.

Response: As mentioned above, the pathologist has now assessed tumor purity as proportion of tumor cells and not by area (Fig3, table 2). We also included in silico tumor purity results from PureCN in table 2 for easier comparison. Lastly, we performed statistical comparison between the pathologist versus in silico purity results by way of correlation (Fig5E-F).

11. In addition to CR1107, CR1121 is also consistent with in silico estimation.

Response: After revising the histopathological estimate of tumor purity as a fraction of tumor cells within the tumor area, we find CR1107 and CR1121 results are not consistent with in silico estimates (Table 2). Some samples display similar purity estimates but no correlation can be found in formal analysis, Figure 5E and F correlations. We also revised the results and discussion sections with this updated analysis.

12. Figure 1C. ABSOLUTE might yield lower purity estimates for higher purity samples (figure 1B), although when the purity is lower, it might be superior than others (figure 1C). Therefore, the conclusion ‘ABSOLUTE yielded lower purity estimates for all samples’ might not be appropriate.

Response: We believe it’s appropriate to conclude that ABSOLUTE analysis results in lower purity estimate compared to the other methods used in this study because of the following reasons:

a. ABSOLUTE purity estimates for most of the organoid samples were low whereas other tools predicted near 100% purity.
b. Correlation between ABSOLUTE and the highest VAFs was positive but weaker than with the other in silico tools.
c. In the mixed samples with ‘known’ cancer cell proportion ranging from 75 to 15%, ABSOLUTE predicted <30% for all samples including the ones with high cancer cell DNA content.

13. P14L249, Figure 5 It is hard to say ‘a trend’ from this data.

Response: We agree and have removed this statement from the manuscript.

14. Figure 3. Please explain the discrepancy of APC mutation of CR1106 between A and B

Response: As discussed above, we cannot rule out selection of cancer cell subclones. However, since no other pathogenic variants could be detected in the CR1106 patient tumor sample either, we believe the tumor purity was too low for accurate variant analysis. In support of this conclusion, the tumor purity estimates for this tumor ranged from 16% with ABSOLUTE to 48% with Sequenza, 59% with PureCN and 20% with pathologist. According to the TCGA guidelines, this is a low purity sample and therefore variant analysis may not be reliable.

We suspect the low purity of the original CR1106 sample and the low abundance of reads covering the APC gene lead to discarding the variant during quality control as not statistically significant. Also, a factor in the detection of low VAFs, particularly when analyzing low purity samples, is the sequencing depth (source below). The sequencing depth must be ultra high to detect low frequency variants in low purity samples. In quality control steps, a minimum depth is used to filter false positive variants. Specifically, in CR1106, variants found in APC in the unfiltered WES had a depth between 1 and 9. Generally mutations with such low sequencing depths will be filtered out in variant calling analysis.

Because of these hurdles, in order to get an accurate characterization of variants in the cancer sample, the sequencing of multiple samples from different regions of the tumor, and/or laser capture microdissection, might be ways to circumvent the problem of tumor heterogeneity and low tumor purity. Unfortunately, this was not possible in our current study. Instead, we used matching organoid samples as controls representing high tumor purity samples and were able to increase the tumor cell content and detect an APC mutation. Tumor-organoid pairs from all other patients had identical pathogenic variants found between them.

https://www.nature.com/articles/s41467-017-01470-y

**Reviewer #2:**

1. In this manuscript Tbeileh et al, explore the use of three in silico tools which utilise WES data to assess the tumour purity in liver mets associated with colorectal cancer. Whilst the premise is a good one, the limited number of samples overall, and the limited number with pathology review make it difficult to draw significant conclusions. I recommend that the authors revise the manuscript and incorporate additional samples in their analysis.

Response:

Thank you for your comments. While we acknowledge the limitations of our small sample size, we believe our results represent an important contribution to understanding the abundance of stroma in metastatic colorectal adenocarcinoma with implications for future genomic and transcriptomic analyses. Namely, TCGA guidelines on minimum tumor purity may not be applicable for most genomic studies and care should be taken when choosing tumor purity assessment methods.

2. Pathologist assessment was performed on only 6 cases. This sample size is rather small and given the inconsistencies in the performance of the 3 tools it is very difficult to make any conclusions. This aspect of the work would be strengthened with a larger number of samples. This would then allow the authors to confirm (or not) and the trends described.

Response: We have now included additional samples (Table 2). In the histopathological comparison we still did not find significant correlation with the variant allele frequencies or with the *in silico* purity results (Fig5E-F). We believe our sample size, while small, is sufficient to report that the selection of tumor purity assessment method is an important consideration for genomic studies and that the TCGA guidelines may not be suitable for all studies. Additionally, our sample set and results show that CRC liver metastases in general have very low tumor purity.

3. The in silico tumour purity vales for Sequenza and PureCN was similar for most samples but was very different for cases PDXO1107 and PDXO1123. It is reasonable to assume (as also mentioned by the authors) that organoid samples which have been enriched for epithelial cells would have a reasonably high purity. Can the authors comment on this difference and explain the low purity levels estimated by PureCN and comment on the accuracy of this tool?

Response: It is in fact interesting that PureCN gives purity estimates lower than 90% on these two samples in comparison with Sequenza. We believe this stems from differences between the two algorithms and highlights the value of testing multiple in silico tools in parallel.

On PureCN’s Github Issues (online documentation), it has been noted that the tool can often yield inaccurate results on cell lines. For this issue in their best practices, they recommend setting the minimum purity to 90% so the algorithm does not consider lower purity solutions. Under these conditions, purities over 90% for these samples will be chosen for the ideal solution since they were of the possible solutions in the PureCN output. They also mention that a fix is in the process of being implemented that includes a number of additional tests to add to their likelihood model such as a test for allelic imbalance (https://github.com/lima1/PureCN/issues/165).

In contrast, one of Sequenza’s main steps includes calculating B-allele frequencies which can be used to infer allelic imbalance in a sample. This difference in evaluating purity may be the cause of such low estimates by PureCN. However, although this problem can be circumvented when using cell lines or organoids by setting the minimum purity to a high value, the same cannot be said for high purity samples that have not been produced through culturing. In this case, a fix is much needed to account for high purity samples.

4. Sample CR1106 was estimated to have 3% tumour and 62% stroma by the pathologist. This was the lowest of all the samples. The VAF for APC of the associated organoid was very high but was not detected at all in the original tumour. Do the authors believe that there is a minimum purity threshold that needs to be met for the tools to be useful?

Response: After the pathologist assessed tumor purity in terms of proportion of tumor cells instead of tumor area, CR1106 purity estimate was 20%. In silico estimates ranges from 16-59% for the original patient sample. All this point toward a low purity tumor which we believe is the reason why the APC mutation was not detected in the patient tumor and only in the matching organoids.

We think setting a purity threshold is context dependent. Minimum purity level will be dependent on the questions and methods used. For whole genome/exome and/or whole transcriptome analyses that aim to characterize the cancer cells within a tumor, we certainly agree with the TCGA minimum purity of 60% and argue that samples may need to be even purer in order to detect low frequency minor variants.

Additionally, what we think this study shows is that the method by which tumor purity is assessed can greatly influence the study outcomes. TCGA guidelines suggest that pre-screening is done by histopathological assessment, whereas we would caution researchers to also perform laser capture microdissection and/or preliminary DNA and/or RNA sequencing followed by in silico tumor purity assessment. For other studies, such as ones aimed at characterizing the stromal transcriptome, prescreening for tumor purity may not be necessary, or low purity samples may be preferred.

5. Fig 4 shows the correlation b/w the VAF of the most prevalent pathogenic variant for each sample and the in silico tumour purity results. Can the authors provide details of the what the variant was for each sample?

Response: Thank you for this comment, we have added the information to Figure 4.

6. Can the authors explain why they chose to use a mix of PDO’s (n=2) and PDXO’s (n=4)? Again, these numbers are small and introduce additional variables.

Response: Reviewer#1 also brought up this concern, see response above. We have included additional explanation in materials and methods, paragraph starting at line 104.
