# Supplementary material for: Metastatic colorectal adenocarcinoma tumor purity assessment from whole exome sequencing data
Source: PLoS One. 2023 Apr 6;18(4):e0271354. doi: 10.1371/journal.pone.0271354 (PMC10079084; doi:10.1371/journal.pone.0271354)
Supplement: Attachment — Submitted filename: Response to Reviewers.docx [file pone.0271354.s002.docx]

Response to Reviewers' comments for the manuscript: **Metastatic colorectal adenocarcinoma purity assessment from whole exome sequencing**

Reviewer #1: I should have mentioned them in the first comments, but I hope the following comments will help to make the manuscript better.

1. *Overall, metastatic specimens had a median tumor purity below 50% whereas the organoids were enriched for cancer cells with purity estimates above 90%.*

As the author added in the revised manuscript, adding ‘according to all computational methods’ would be better than just ‘overall’. Where is the value of ‘90%’ described in the results? Actually, the average of Figure 1B is 70%.

**Our response**: We thank the reviewer for pointing out this error. We neglected to update this part of the abstract when we re-analyzed data for our previous resubmission. We have now modified the abstract text to show the purity median for all patient samples and for all organoids (across all computational methods), line 37. Median tumor purity results are given separately for each computational tool in the results section. Thus we have harmonized the description of the analysis results in the abstract with the analysis in the body of the manuscript.

2. *In line with this, VAFs of oncogenes and tumor suppressor genes were undetectable or low in patient tumors, but high in matching organoid cultures.*
   Since some of the VAFs are 100% in Figure 4, it would be better to put ‘in some cases’ in this sentence.

**Our response:** We are unsure where in this sentence the reviewer wishes to include ‘in some cases’. We slightly modified the sentence in the abstract and hope it is clearer now, line 40.

3. In Figure 5F: the legend of the x-axis, in silico tumor purity % (PureCN) would be better than just PureCN.

**Our response:** We have modified the figure legend as suggested.
